# Multifunctional Role of Bcl-2 in Malignant Transformation and Tumorigenesis of Cr(VI)-Transformed Lung Cells

**DOI:** 10.1371/journal.pone.0037045

**Published:** 2012-05-29

**Authors:** Djordje Medan, Sudjit Luanpitpong, Neelam Azad, Liying Wang, Bing-Hua Jiang, Mary E. Davis, John B. Barnett, Lan Guo, Yon Rojanasakul

**Affiliations:** 1 Department of Pharmaceutical Sciences, West Virginia University, Morgantown, West Virginia, United States of America; 2 Department of Pharmaceutical Sciences, Hampton University, Hampton, Virginia, United States of America; 3 Pathology and Physiology Research Branch, National Institute for Occupational Safety and Health, Morgantown, West Virginia, United States of America; 4 Department of Pathology, Anatomy and Cell Biology, Thomas Jefferson University, Philadelphia, Pennsylvania, United States of America; 5 Department of Physiology and Pharmacology, West Virginia University, Morgantown, West Virginia, United States of America; 6 Department of Immunology, Microbiology and Cell Biology, West Virginia University, Morgantown, West Virginia, United States of America; 7 Mary Babb Randolph Cancer Center, West Virginia University, Morgantown, West Virginia, United States of America; West Virginia University School of Medicine, United States of America

## Abstract

B-cell lymphoma-2 (Bcl-2) is an antiapoptotic protein known to be important in the regulation of apoptosis in various cell types. However, its role in malignant transformation and tumorigenesis of human lung cells is not well understood. We previously reported that chronic exposure of human lung epithelial cells to the carcinogenic hexavalent chromium Cr(VI) caused malignant transformation and Bcl-2 upregulation; however, the role of Bcl-2 in the transformation is unclear. Using a gene silencing approach, we showed that Bcl-2 plays an important role in the malignant properties of Cr(VI)-transformed cells. Downregulation of Bcl-2 inhibited the invasive and proliferative properties of the cells as well as their colony forming and angiogenic activities, which are upregulated in the transformed cells as compared to control cells. Furthermore, animal studies showed the inhibitory effect of Bcl-2 knockdown on the tumorigenesis of Cr(VI)-transformed cells. The role of Bcl-2 in malignant transformation and tumorigenesis was confirmed by gene silencing experiments using human lung carcinoma NCI-H460 cells. These cells exhibited aggressive malignant phenotypes similar to those of Cr(VI)-transformed cells. Knockdown of Bcl-2 in the H460 cells inhibited malignant and tumorigenic properties of the cells, indicating the general role of Bcl-2 in human lung tumorigenesis. Ingenuity Pathways Analysis (IPA) revealed potential effectors of Bcl-2 in tumorigenesis regulation. Additionally, using IPA together with ectopic expression of p53, we show p53 as an upstream regulator of Bcl-2 in Cr(VI)-transformed cells. Together, our results indicate the novel and multifunctional role of Bcl-2 in malignant transformation and tumorigenesis of human lung epithelial cells chronically exposed to Cr(VI).

## Introduction

Lung cancer is the leading cause of cancer mortality worldwide. While the etiology of lung cancer caused by various agents including cigarette smoke, air pollution, and heavy metals has been established [1], [2], the underlying mechanisms of tumorigenesis are not well understood. Current research indicates that long-term exposure to inhaled carcinogens has the greatest impact on the risk of lung cancer. Cr(VI)-containing compounds are ubiquitous carcinogens associated with the incidence of lung cancer in humans. Several epidemiological studies in the last few decades have associated exposure to Cr(VI) with the induction of lung cancer in workers in various occupational settings [3]–[6]. Cr(VI) compounds are also present in cigarette smoke, automobile emissions, and are widespread in the environment, e.g., Cr(VI)-contaminated water [7], [8]. In the United States, an air quality survey indicated that people in several residential areas are exposed to particulate airborne chromium at concentrations exceeding 100 times the chronic toxicity benchmark, which set at 0.016 mg/m^3^ from the critical study used as the basis for Environmental Protection Agency's reference concentration for Cr(VI) particulates [9], [10]. Therefore, in addition to occupational exposure, environmental chromium at high concentrations is an emerging concern for its associated long-term carcinogenic effect on the lungs.

Cr(VI)-containing compounds have been designated as Class I human carcinogens by IARC based on epidemiological data and a large body of knowledge showing that they are mutagenic and genotoxic [11]. However, animal studies have yielded inconsistent or negative results [12]–[14] due to genetic variations or other predisposing factors that are not well understood. The lack of good animal models has hindered the efforts to identify the mechanisms of Cr(VI)-induced tumorigenesis. Therefore, even though Cr(VI) compounds have been identified as human carcinogens, the underlying mechanisms remain elusive. To date, most Cr(VI) tumorigenesis studies have focused on short-term or acute exposure effects; however, tumorigenesis is a long-term process requiring chronic exposure to carcinogens. To mimic the pathological process, we utilized a chronic exposure model of human lung epithelial BEAS-2B cells and examined the long-term effects of Cr(VI) exposure and the role of Bcl-2 in the cell transformation and carcinogenic process. The BEAS-2B cells were used because they exhibit similar characteristics and cellular responses to carcinogen as the primary or normal lung cells. They are also non-tumorigenic and can be grown continuously in the culture, thus allowing long-term exposure studies.

**Figure 1:**
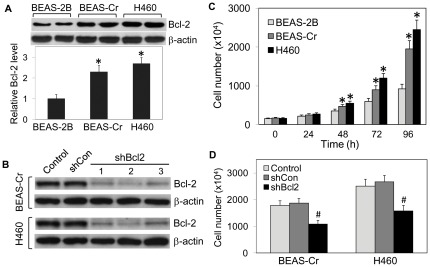
Bcl-2 expression and effect of Bcl-2 knockdown on cell growth. (*A*) Endogenous Bcl-2 levels in BEAS-2B, BEAS-Cr and H460 cells determined by Western blot analysis. Densitometry was performed to determine the relative Bcl-2 levels after reprobing the membrane with β-actin antibody. (*B*) Bcl-2 knockdown experiments were performed in BEAS-Cr and H460 cells by infecting the cells with Bcl-2 shRNA (shBcl-2) viral particles or control shRNA (shCon) particles as described under *Materials and Methods*. Clonal selection and Bcl-2 expression were performed to identify mutants with downregulated Bcl-2. (*C*) BEAS-2B, BEAS-Cr and H460 cells (1×10^5^ cells) were seeded on 60-mm cell culture dishes and were incubated at 37°C in a 5% CO_2_ incubator. At the indicated times, cells were trypsinized and analyzed for cell number using an electronic cell counter. (*D*) Effect of Bcl-2 on cell growth of the respective cell lines and their mutants. Cells (1×10^5^ cells) were seeded and incubated at 37°C for 96 h, after which they were analyzed for cell number. Values are means (± SD) (*n* = 4). *P<0.05 versus passage-control BEAS-2B cells. ^#^ *P*<0.05 versus the respective BEAS-Cr and H460 cells.

**Figure 2 pone-0037045-g002:**
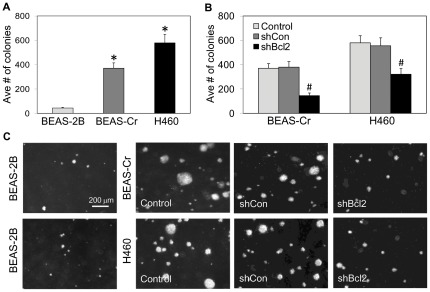
Effect of Bcl-2 knockdown on colony formation of BEAS-Cr and H460 cells. (*A*) BEAS-2B, BEAS-Cr and H460 cells (3×10^4^ cells) were seeded on 0.5% agar plates and incubated at 37°C in a 5% CO_2_ incubator. After 2 weeks, colony formation capacity of the cells was scored under a light microscope. (*B*) Effect of Bcl-2 on colony formation capacity of the respective cell lines and their mutants. Cells (3×10^4^ cells) were seeded on soft agar plates and the colonies were scored after 2 weeks. (*C*) Representative micrographs of colonies formed by the cell lines and mutants on soft agar are shown. Values are means (± SD) (*n* = 4). *P<0.05 versus passage-control BEAS-2B cells. ^#^ *P*<0.05 versus the respective BEAS-Cr and H460 cells.

**Figure 3 pone-0037045-g003:**
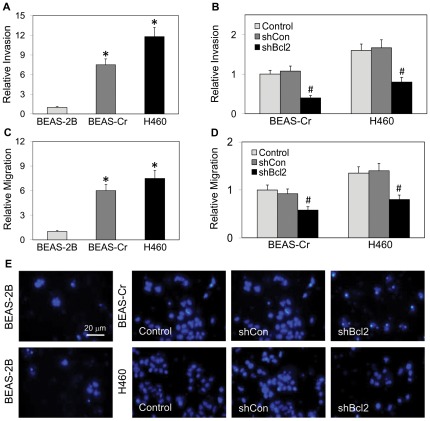
Effect of Bcl-2 knockdown on cell invasion and migration of BEAS-Cr and H460 cells. (*A*) BEAS-2B, BEAS-Cr and H460 cells (1×10^5^ cells) were added to Transwell® inserts coated with Matrigel® and incubated for 24 h. Invading cells were stained and counted under a light microscope. Plots show relative invasion of BEAS-2B, BEAS-Cr and H460 cells. (*B*) Effect of Bcl-2 on cell invasion of the respective cell lines and their mutants. Experiments were repeated with the indicated cell lines and analyzed for cell invasion. (*C*) Confluent monolayers of BEAS-2B, BEAS-Cr and H460 cells were wounded, and the cells were allowed to migrate for 24 h. Wound space was visualized by light microscopy and analyzed by comparing the relative change in wound space as compared to control cell monolayers. (*D*) Effect of Bcl-2 on cell migration of the respective cell lines and their mutants. Cells were wounded and analyzed for cell migration over a 24 h period. (*E*) Representative micrographs of cells stained for invasion are shown. Values are means (± SD) (*n* = 4). *P<0.05 versus passage-control BEAS-2B cells. ^#^ *P*<0.05 versus the respective BEAS-Cr and H460 cells.

**Figure 4:**
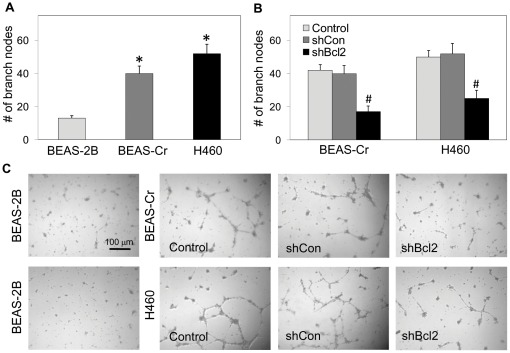
Effect of Bcl-2 knockdown on angiogenic activity of BEAS-Cr and H460 cells. (*A*) BEAS-2B, BEAS-Cr, and H460 cell supernatants were incubated with HUVEC cells, and endothelial capillary tube formation was detected under a light microscope. The number of nodes formed by the tubes were scored and plotted. (*B*) Effect of Bcl-2 knockdown on angiogenic activity of the respective cell lines. Experiments were performed with the indicated cell lines and analyzed for endothelial tube formation as described. (**C**) Representative micrographs of tube formation are shown. Values are means (± SD) (*n* = 4). *P<0.05 versus passage-control BEAS-2B cells. ^#^ *P*<0.05 versus the respective BEAS-Cr and H460 cell supernatants.

**Figure 5:**
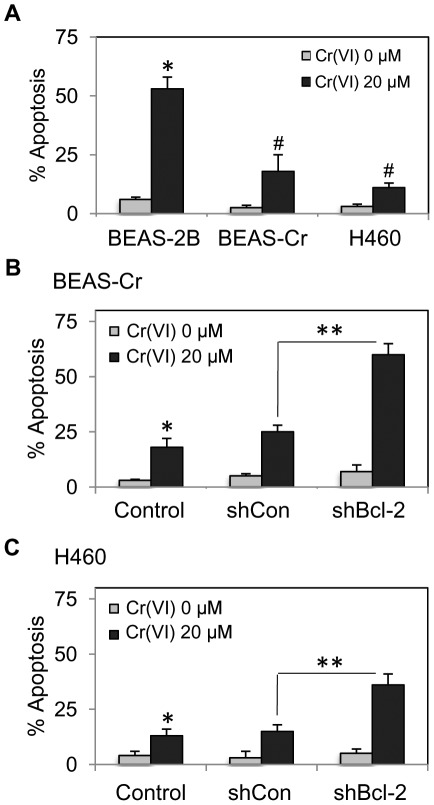
Apoptosis response to Cr(VI) treatment in BEAS-2B, BEAS-Cr, and H460 cells. (*A*) BEAS-2B, BEAS-Cr and H460 cells were treated with or without Cr(VI) (20 µM) for 12 h and apoptosis was determined by Hoechst 33342 assay. (*B*) and (*C*) Effect of Bcl-2 knockdown on Cr(VI)-induced apoptosis of the respective cell lines and their mutants. Values are means (± SD) (*n* = 4). *P<0.05 versus non-treated control. ^#^ *P*<0.05 versus Cr(VI)-treated passage-control BEAS-2B cells. **P<0.05 versus Cr(VI)-treated respective BEAS-Cr and H460 cells.

**Figure 6:**
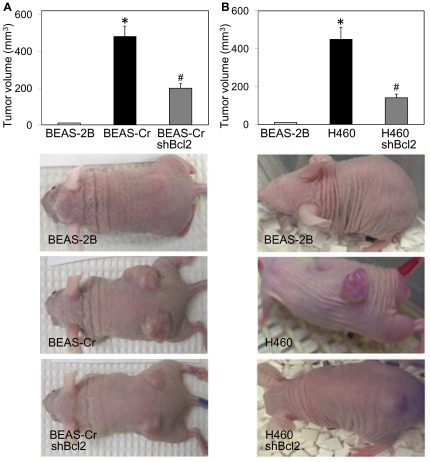
Effect of Bcl-2 knockdown on tumor-associated properties *in vivo*. (*A*) Mice were injected subcutaneously with 1×10^6^ passage-control BEAS-2B, BEAS-Cr, or shBcl-2 BEAS-Cr cells. Tumor formation was determined at 14 d post-injection. Representative photographs are shown. (*B*) Mice were similarly injected with control BEAS-2B, H460, or shBcl-2 H460 cells. Tumor formation and representative micrographs at 14 d post-injection are shown. Data are means (± SD) (*n* = 4). *P<0.05 versus passage-control BEAS-2B cells. ^#^ *P*<0.05 versus the respective BEAS-Cr and H460 cells.

**Figure 7:**
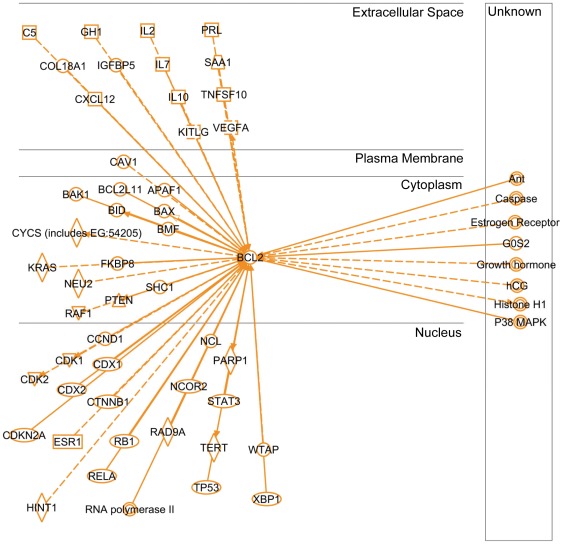
Ingenuity Pathways Analysis software output for the Bcl-2-interactome. IPA query for the Bcl-2-interactome. The interactome was filtered for interactions reported in humans only and presented by cellular localization. 56 molecules were reported to interact either directly (solid lines) or indirectly (dash lines). Arrow direction indicates direction of functionality.

**Figure 8:**
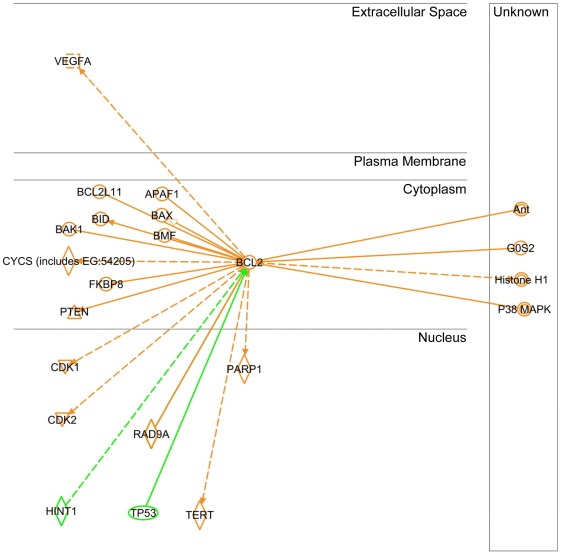
Ingenuity Pathways Analysis software output for the downstream components of human Bcl-2-interactome. Twenty molecules of downstream components were reported to date and were labeled in orange, while upstream molecules were labeled in green.

**Figure 9:**
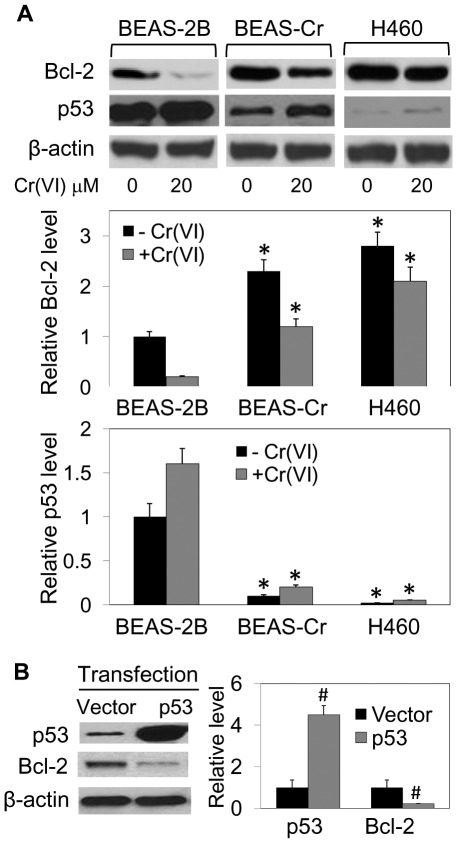
Bcl-2 and p53 expression in BEAS-2B, BEAS-Cr and H460 cells. (*A*) Cells were either left untreated or treated with Cr(VI) (20 µM) for 12 h. Cell lysates were prepared and analyzed for Bcl-2 and p53 by Western blotting. β-actin was used as a loading control. Densitometry was performed to determine the relative levels of Bcl-2 and p53 compared to β-actin. Representative blots are shown. (*B*) BEAS-Cr cells were transiently transfected with pcDNA (vector) or p53 plasmid, and Bcl-2 and p53 expression levels were determined by Western blotting. Values are means (± SD) (*n* = 4). *P<0.05 versus BEAS-2B controls. ^#^ *P*<0.05 versus vector-transfected control.

Bcl-2 is an anti-apoptotic protein known to be important in the regulation of apoptosis induced by various agents including Cr(VI) [15], [16]. We have previously shown that chronic exposure of lung epithelial cells to Cr(VI) causes an upregulation of Bcl-2 [17]. However, its role in malignant transformation and tumorigenesis is unknown. Several human small and non-small lung cancer cell lines and tumor specimens have been shown to overexpress Bcl-2 [18]–[20]. This protein has also been shown to be upregulated in many forms of cancer, including 90% of colorectal cancer, 70% of breast cancer, and 30–60% of prostate cancer [21], [22]. Accordingly, anti-Bcl-2 strategies have been widely developed as novel cancer therapy for various malignancies [21]–[23]. While these studies suggest the potential role of Bcl-2 in Cr(VI)-induced tumorigenesis, direct evidence is lacking. In this study, we used a gene silencing approach to knockdown Bcl-2 in Cr(VI)-transformed cells and studied its effects on cancer-associated properties including cell growth, apoptosis, invasion, colony formation, and angiogenesis. We also studied the tumorigenic effect of Cr(VI)-transformed cells *in vivo* and examined the role of Bcl-2.

## Materials and Methods

### Ethics statement

All animal procedures were conducted in accordance with the guidelines for the Use and Care of Laboratory Animals and approved by the West Virginia University Institutional Animal Care and Use Committee (WVU IACUC).

### Cell culture and reagents

Human lung epithelial BEAS-2B, human non-small (large) cell lung cancer H460 cells derived from patient pleural effusion, and human umbilical vein endothelial cells (HUVEC) were obtained from American Type Culture Collection (ATCC; Manassas, VA). BEAS-2B cells were cultured in DMEM medium containing 5% fetal bovine serum (FBS), 2 mM L-glutamine, 100 units/ml penicillin/streptomycin. H460 cells were cultured in RPMI 1640 medium containing 10% FBS. HUVEC cells were cultured in F12K medium supplemented with 10% FBS, 0.1 mg/ml heparin sulfate, 0.05 mg/ml endothelial cell growth factor supplement (BD Biosciences, San Jose, CA), 100 units/ml penicillin/streptomycin. All cells were cultured in a 5% CO_2_ environment at 37°C. Sodium dichromate (Na_2_Cr_2_O_7_.2H_2_O) [Cr(VI)] was obtained from Sigma (St. Louis, MO). Lipofectamine was obtained from InVitrogen (Carlsbad, CA). Hoechst 33342 was from Molecular Probes (Eugene, OR).

### Chronic Cr(VI) exposure

Subconfluent cultures of BEAS-2B cells (1×10^5^ cells) in 6-well plates were continuously exposed to 5 µM Cr(VI). Cr(VI)-containing culture medium was changed every 3 to 4 days and the cells were passaged weekly at preconfluent (∼80%) densities. The Cr(VI)-treated cells are designated as BEAS-Cr cells so as to distinguish them from the parental BEAS-2B cells. Parallel cultures grown in Cr(VI)-free medium provided passage-matched controls. After 24 weeks of exposure, Cr(VI)-treated cells were cultured in normal medium and their malignant transformation and tumorigenic properties were assessed as described below.

### Cell growth assay

Cells (1×10^5^ cells) were seeded on 60-mm cell culture dishes. At specific times after the incubation, cells were trypsinized and analyzed for cell number using a Countess® automated cell counter (InVitrogen, Carlsbad, CA).

### Soft agar colony formation assay

Soft agar assay was performed as previously described with minor modifications [24]. The cell lines studied (3×10^4^ cells) were mixed with tissue culture medium containing 0.5% agar to result in a final agar concentration of 0.33%. The cell suspension (1.5 ml) was plated into 60-mm dishes coated with 7 ml of 0.5% agar into culture medium. After 2 weeks, the average number of colonies with a size of more than 50 cells was scored under a light microscope.

### Migration and invasion assays

Cell migration was determined by wound assay as detailed previously [25]. Briefly, monolayer of cells was cultured in 24-well plate, and a wound space was made with 1 mm width tip. After rinsing with PBS, the cell monolayers were allowed to migrate for 24 h. Cell migration was measured by light microscopy and quantified by calculating the percentage of change in the wound space as previously described [25]. Relative cell migration was calculated by dividing the percentage change in the wound space of transformed cells by that of the control cells in each experiment. Invasion assays were performed using Transwell® chambers coated with Matrigel® (50 µl per filter) (BD Biosciences, NJ) according to the manufacturer's protocol. Medium containing 5% FBS was used as a chemo-attractant. After 24 h incubation, cells that invaded to the underside of chamber were stained with Hoechst 33342.

### Angiogenesis tube formation assay

Growth factor-reduced Matrigel® was placed in 24-well plates (150 µl/well) and allowed to set at 37°C for 30 min. HUVEC cells (4×10^4^cells) suspended in 2.5% dialyzed FBS medium were incubated with cell-primed supernatants from epithelial cells (0.5×10^6^ cells/6-well plate) at the ratio of 1 to 1, and added to the Matrigel®-coated plates. Tube formation of HUVEC cells was observed and photographed using a phase contrast microscope after 24 h. The number of nodes formed was scored from at least five different fields for each well.

### Apoptosis assay

Apoptosis was determined by Hoechst 33342 DNA fragmentation assay. Briefly, cells were incubated with 10 µg/ml of Hoechst 33342 for 30 min and analyzed for apoptosis by scoring the percentage of cells having intensely condensed chromatin and/or fragmented nuclei by fluorescence microscopy (Leica Microsystems, Bannockburn, IL). Approximately 1,000 nuclei from ten random fields were analyzed for each sample. The apoptotic index was calculated as the percentage of cells with apoptotic nuclei over total number of cells.

### Generation of Bcl-2 knockdown clones

Lentiviral transduction particles carrying short hairpin RNA (shRNA) sequence against human Bcl-2 (5′-CCGGGTGATGAAGTACATCCATTATCTCGAGATAATGGATGTACTTCATCA CTTTTG-3′) and control non-target sequence (5′-CCGGCAACAAGATGAAGAGCACCAACT CGAGTTGGTGCTCTTCATCTTGTTGTTTTT-3′) were used to downregulate Bcl-2 expression in BEAS-Cr and H460 cells. The viral vectors were obtained from Sigma (Cat # SHVRS-NM_000633 and SHC002V, respectively) and were used according to the manufacturer's protocol at the multiplicity of infection of 1.5. For generation of stable clones, cells were cultured for 14 days with puromycin at 1 µg/ml concentration. Resistant clones expressing varying levels of Bcl-2 were isolated using cloning cylinders (Bellco Glass, Vineland, NJ) and transferred for expansion and analysis by Western blotting.

### Western blotting

Preparation of cell lysates and Western blotting were performed as described previously [16]. We obtained antibodies for Bcl-2, β-actin, and peroxidase-conjugated secondary antibodies from Santa Cruz Biotechnology (Santa Cruz, CA). The immune complexes were detected by chemiluminescence and quantified using analyst/PC densitometry software (Bio-Rad Laboratories, Hercules, CA).

### *In vivo* tumorigenesis

Male athymic (nu/nu) nude mice obtained from Jackson Laboratory (Bar Harbor, ME) were housed under pathogen-free conditions and fed an autoclaved diet and water ad libitum. To establish tumor xenografts in mice, BEAS-2B, BEAS-Cr, H460 and Bcl-2 knockdown cells (1×10^6^ cells) were suspended in 1∶1 medium mixed with Matrigel® and were injected subcutaneously on the left and right flanks of each mouse. Tumor size was measured at 14 days post-injection by using a caliper, and the tumor volume was determined using the formula: 0.5238×L (length)×W (width)×H (height) of the tumor. All procedures were conducted in accordance with the guidelines for the Use and Care of Laboratory Animals and approved by the West Virginia University Institutional Animal Care and Use Committee (WVU IACUC).

### Bcl-2-interactome analysis

The Ingenuity Pathways Analysis (IPA) software (Ingenuity Systems, Redwood City, CA) was used to analyze the currently published Bcl-2 interactome. The Ingenuity database is the largest curated database of previously published findings on mammalian biology from the public literature (i.e., MEDLINE). Reports on individual studies of genes in human, mouse or rat were first identified from peer-reviewed publications, and findings were then encoded into ontology by content and modeling experts. Network analysis using the knowledge base was used to further identify direct interactions between mammalian orthologues. The Bcl-2-interactome is a graphical representation of its published molecular relationships. Molecules are represented as nodes, and the biological relationship between two nodes is represented as an edge (line). Nodes are displayed using various shapes that represent the functional class of the gene product. Edges are displayed with various labels that describe the nature of the relationship between the nodes.

### Statistical analysis

The data were expressed as means ± SD of three or more independent experiments. Statistical analysis was performed using two-tailed and paired Student's *t*-test. *P* values less than 0.05 were considered statistically significant and indicated by an asterisk.

## Results

### Bcl-2 expression and cell growth

Abnormal cell growth is a key characteristic of cancer cells. We determined the growth characteristic of Cr(VI)-transformed cells (BEAS-Cr) generated by chronically exposing non-carcinogenic BEAS-2B cells to Cr(VI) over 24 weeks as described under *Materials and Methods*. To determine the role of Bcl-2 in the process, the transformed BEAS-Cr cells were first analyzed for Bcl-2 expression and cell growth characteristics. Passage-matched BEAS-2B cells and human lung cancer H460 cells were used as a negative and positive control, respectively. Figure 1A shows that BEAS-Cr cells exhibited a higher level of Bcl-2 protein expression as compared to passage-control BEAS-2B cells, but comparable to H460 cells. To study the role of Bcl-2 in Cr(VI)-induced transformation, mutant cell lines exhibiting stably downregulated Bcl-2 were generated from BEAS-Cr and H460 cells using RNA interference and clonal selection. Figure 2B shows that maximum Bcl-2 downregulation in BEAS-Cr mutants (85%) was achieved in clone 2, whereas maximum reduction in H460 mutants (75%) was obtained in clone 1, which were used in subsequent studies. Figure 1C shows that as compared to control BEAS-2B cells, BEAS-Cr and H460 cells exhibited a significantly higher growth rate which was observed as early as 48 h post-seeding. By 96 h, the growth rates of BEAS-Cr and H460 cells were more than double that of the control BEAS-2B cells. Knockdown of Bcl-2 in BEAS-Cr and H460 cells reduced the growth rates by about 50% at 96 h (Figure 1D).

### Colony formation

Soft agar colony formation assays were performed to assess the relative colony forming activities of the cells. BEAS-2B, BEAS-Cr, and H460 cells were subjected to colony formation assay by growing them on agar plates so as to assess anchorage-independent growth. After 2 weeks, significant colony formation was observed in BEAS-Cr cells with a 7-fold increase as compared to passage-control BEAS-2B cells which also formed very few, slow growing colonies (Figure 2A and C). The H460 lung cancer cells exhibited the highest colony formation capacity, out-growing the BEAS-2B cells by approximately 8-fold and BEAS-Cr cells by approximately 1.5-fold. Next, the effect of Bcl-2 knockdown on colony formation capacity was evaluated. The BEAS-Cr mutant exhibited approximately 60% reduction in colony formation, while a 40% reduction was observed in the H460 mutant as compared to their respective controls (Figure 2B and C).

### Invasion and migration

We next investigated the relative aggressive-malignant phenotypes of the cells using the Transwell® invasion and wound migration assays. The H460 cells showed the highest invasion capacity with BEAS-Cr cells invading at the rate of 70% relative to H460, and BEAS-2B invading at 15% (Figure 3A and E). A 7-fold increase in invasion rate was observed in BEAS-Cr as compared to BEAS-2B cells. The highest migration capacity was observed in H460 cells followed by BEAS-Cr cells, at approximately 75% of H460 cells, and finally, BEAS-2B cells, at less than 10% of H460 cells (Figure 3C). Knockdown of Bcl-2 in BEAS-Cr and H460 cells substantially reduced their invasion and migration capacities as compared to controls (Figure 3B, D and E).

### Angiogenesis

Angiogenesis is an essential step in tumor growth. We determined whether Cr(VI)-transformed cells possess an angiogenic activity and whether Bcl-2 regulates this activity by performing vascular endothelial tube formation assay. HUVEC cells were incubated with cell-primed supernatants from BEAS-Cr, H460, or control BEAS-2B cells in Matrigel®-coated plates, and analyzed for endothelial capillary tube formation. Figure 4A and C shows that BEAS-Cr and H460 cell supernatants induced a higher number of branch nodes and a more complex pattern of endothelial tube formation as compared to control BEAS-2B cell supernatants. This result indicates an increase in pro-angiogenic activity of Cr(VI)-transformed cells which may be important in Cr(VI)-induced tumorigenesis that has not been reported. Downregulation of Bcl-2 in BEAS-Cr and H460 cells inhibited their angiogenic activity as compared to controls (Figure 4B and C). This finding indicates a novel and positive regulatory role of Bcl-2 in angiogenesis.

### Apoptosis resistance

Resistance to apoptosis is a foundation of neoplastic evolution and a key characteristic of cancer cells. We tested whether Cr(VI)-transformed cells exhibited apoptosis-resistant phenotype and whether Bcl-2 plays a role. BEAS-Cr, H460, or control BEAS-2B cells were treated with Cr(VI) and analyzed for apoptosis. Figure 5A shows that Cr(VI) treatment markedly induced apoptosis of control BEAS-2B cells, whereas it had substantially lesser effect in BEAS-Cr and H460 cells. Knockdown of Bcl-2 in BEAS-Cr and H460 cells reversed their apoptosis resistance as compared to their respective controls (Figure 5B and C). The mechanistic similarity between BEAS-Cr and H460 cells suggests that Bcl-2 is a general feature of apoptosis resistance to Cr(VI).

### Effect of Bcl-2 knockdown on tumor formation *in vivo*

To assess the potential tumorigenicity of Cr(VI)-transformed cells and the role of Bcl-2 in the process, experiments were performed using a xenograft mouse model. BEAS-Cr and H460 cells and their Bcl-2 knockdown mutants were subcutaneously injected into nude mice. At one week after the injection, small tumors were formed at the injection site in mice receiving BEAS-Cr and H460 cells, whereas mice receiving passage-control BEAS-2B cells did not develop tumors. At 14 days post-injection, large tumors were found in the BEAS-Cr (Figure 6A) and H460 (Figure 6B) mice, whereas no or very small lumps were observed in the BEAS-2B control mice. Mice receiving Bcl-2 knockdown mutants, either BEAS-Cr or H460, showed a substantial reduction in tumor volume as compared to their respective controls (Figure 6A and B). These results indicate the role of Bcl-2 in tumorigenesis of Cr(VI)-transformed cells. The inhibition of tumor growth in H460 mutant mice also implicates the general role of Bcl-2 in the tumorigenesis of lung cancer cells.

### Bcl-2-interactome analysis

In order to establish a strategy towards a better understanding of the mechanism(s) involved in Bcl-2's contribution to tumorigenesis, we performed an extensive PubMed database search for possible molecular targets. The initial query for Bcl-2 returned 33,970 hits! We realized that other approaches may need to be evaluated given the complexity of Bcl-2 cellular role(s). Following the analysis of available options, we turned to Ingenuity Pathways Analysis (IPA). IPA integrates the primary literature into easily searched and visualized networks allowing for orders of magnitude faster evaluation of cellular signaling cascades. The initial IPA query of Bcl-2's interactions (IPA v8.6) returned 741 hits. In order to make the network more relevant to our model, we filtered for only those interactions reported in humans (Figure 7). This reduced the interaction network to 56 molecules organized by cellular localization (Bcl-2-interactome). Given that we selectively targeted Bcl-2 and observed significant consequences on tumor-associated properties, we asked IPA to map only the downstream components of human Bcl-2-interactome known to date (Figure 8). The query returned 20 molecules. As we progressed from the initial overwhelming complexity of data to the more manageable and relevant Figure 6B, the current mechanistic evidence of Bcl-2's role in tumorigenesis emerged, as did a new approach to scientific query.

### Regulation of Bcl-2 by p53

It is likely that multiple genes and signaling pathways are involved in the different effects of Bcl-2. In an effort to understand the mechanism of Bcl-2 regulation in Cr(VI)-transformed cells, we investigated the potential role of p53 since it is known to regulate several proteins in the Bcl-2 family [26] and it is shown by IPA a direct upstream regulator of Bcl-2 (Figure 8). Cells were treated with or without Cr(VI) and analyzed for Bcl-2 and p53 protein levels by Western blotting. In non-treated cells, Bcl-2 expression was substantially lower in the BEAS-2B cells as compared to BEAS-Cr and H460 cells, whereas p53 expression was higher in BEAS-2B cells (Figure 9A). The inverse relationship between Bcl-2 and p53 was also observed in Cr(VI) treated cells across the cell lines tested, indicating the potential negative regulation of Bcl-2 by p53. To test the direct role of p53 in Bcl-2 regulation in Cr(VI)-transformed cells, BEAS-Cr cells were ectopically transfected with p53, and Bcl-2 expression was evaluated by Western blotting. Figure 9B shows that as compared to vector-transfected control, the p53-transfected cells exhibited a substantially lower Bcl-2 expression, indicating p53 as a negative regulator of Bcl-2 in the cell system.

## Discussion

Although Cr(VI) compounds have been identified as human carcinogens, the underlying mechanisms of tumorigenesis remain unclear. In this study, we reported a combined *in vitro*-*in vivo* model for Cr(VI) tumorigenesis studies using chronically exposed human bronchial epithelial BEAS-2B cells and a mouse xenograft model. Bronchial epithelial cells were chosen in this study because they are a key target for Cr(VI)-induced tumorigenesis. BEAS-2B cells have been widely used in the literature to define conditions under which various agents and oncogenes cause neoplastic transformation [27]–[29]. They have been shown to exhibit similar characteristics and cellular responses to carcinogens as primary or normal lung cells [30]–[32]. Although BEAS-2B cells have a mutated p53^Ser47^ gene, previous studies have shown that this mutation does not affect its growth suppressing and apoptotic function, which is controlled by p53^Ser15^ and is normal in the BEAS-2B cells [30], [31], [33]. Inclusion of the passage-control BEAS-2B cells and bronchial carcinoma H460 cells into direct comparisons was critical when assessing the significance of tumorigenic properties in the evolved BEAS-Cr phenotype. H460 cells are a well established model for *in vitro* and *in vivo* mechanistic studies of lung tumorigenesis and therefore represent a positive control. For BEAS-Cr cells, a significant growth increase was observed as early as 48 h and at 96 h the growth rate was double that of control BEAS-2B cells (Figure 1C). H460 cells proliferated even faster, at approximately 2.5 times that of BEAS-2B cells.

Anchorage-dependent growth is a typical feature of normal cells and controls cell division [34], [35], however when cells are transformed they lose this property. Anchorage-independent growth has been widely correlated with the tumorigenicity and invasiveness of several cancer cell types [36]. Soft agar assay is a stringent test to study the ability of cells to undergo anchorage-independent growth. The number of colonies formed by BEAS-Cr cells was about 7 times more than that in control BEAS-2B cells, confirming the carcinogenic potential of Cr(VI)-transformed cells (Figure 2A and C). H460 cells formed colonies at even higher rate, exceeding BEAS-Cr cells by about 30%. Interestingly, bronchial epithelial BEAS-2B cells obtained at autopsy of non-cancerous individuals also formed a small number of slow growing colonies on soft agar, consistent with previous reports [28], [35]. Additionally, the American Type Culture Collection indicates that the BEAS-2B cell line forms colonies in semisolid medium but is non-tumorigenic in immunosuppressed mice. Therefore, the colony formation observed in BEAS-2B cells is due to the indigenous properties of the cell and not because of their tumorigenic property.

Various carcinogenic properties representing the hallmarks of cancer were further assessed in this study [36]. Cr(VI)-transformed cells were first characterized for their invasive and migratory properties and compared to those of established lung cancer H460 cells. Invasion and migration were shown to increase significantly as compared to passage-control BEAS-2B cells (Figure 3A and C). Previous studies indicate that BEAS-2B are non-tumorigenic, aneuploid cells but can undergo squamous differentiation in response to serum and transforming growth factor-β [37]. However, since passage-control BEAS-2B cells showed no phenotypic changes or malignant behavior, it can be concluded that BEAS-Cr cells are Cr(VI)-transformed BEAS-2B cells and not BEAS-2B cells that have undergone differentiation and show altered phenotype due to continued passaging. The observation of malignant transformation induced by long-term Cr(VI) exposure is consistent with previous reports [38], [39]. In addition, the results of this study indicate that long-term exposure to Cr(VI) also increased the proangiogenic activity of human lung epithelial cells (Figure 4A and C). Since angiogenesis is required for tumor growth, this new finding supports the *in vivo* tumorigenicity of Cr(VI)-transformed cells shown in Figure 6A.

Bcl-2 is known to be a key anti-apoptotic protein involved in the regulation of apoptosis [15], [16]. In the present study, we demonstrated that Bcl-2 dysregulation mediated BEAS-Cr and H460 cells to acquire apoptosis resistance to Cr(VI) (Figure 5A–D). We also found that p53 is a likely upstream regulator of Bcl-2 in this cell system since its ectopic expression downregulated the Bcl-2 level (Figure 9B). This result is good agreement with previous reports showing the repression of Bcl-2 gene expression by p53 [40]–[42] and with Ingenuity Pathways Analysis showing p53 (TP53 gene) as an upstream regulator of Bcl-2 (Figure 8). The mechanism by which p53 regulates Bcl-2 is incompletely understood, but is believed to involve transcriptional regulation since the gene promotor of Bcl-2 contains a p53-negative response element [42].

While the role of Bcl-2 in apoptosis regulation is well established, its role in tumorigenesis is unclear. To determine this relationship, gene knockdown experiments were performed to assess the Bcl-2's contribution to tumorigenic phenotype of BEAS-Cr and H460 cells. Our results showed that Bcl-2 knockdown led to a significant reduction of all malignant properties evaluated (Figures 1–4). Furthermore, our *in vivo* data showed that BEAS-Cr cells formed tumors in nude mice and that Bcl-2 knockdown substantially inhibited the tumor formation (Figure 6A). This mechanistic feature was nearly identical in human lung cancer H460 derived tumors (Figure 6B), indicating a potential shared mechanism between laboratory Cr(VI)-induced tumorigenesis and human lung cancer.

Given that we were interested in the mechanisms involved Bcl-2's contribution to tumorigenesis, we performed an IPA with the main focus on Ingenuity's output of known downstream effectors of Bcl-2-interactome (Figure 8). Did any of the molecules listed have a link to regulation of tumor-associated properties such as proliferation, transformation, migration, invasion and colony formation? Analysis of Ingenuity Knowledge Base (IKB) strongly suggests YES. Out of all downstream Bcl-2 effectors, nucleus-localized constituents strongly argue that Bcl-2's role in tumorigenesis exceeds that of apoptosis regulation in line with our observations. Cyclin-dependent kinases 1 and 2 (CDK1&2) regulate cell cycle progression, cellular motility and proliferation according to IKB summary of over 6,000 categorized literature findings. Their role in tumorigenesis is well established. Next, telomerase (TERT) is an established positive regulator of cell proliferation, immortalization and transformation. Its role in tumorigenesis is widely accepted. Of interest, RAD9A, implicated in the regulation of colony formation also appears in the IPA's output. Relative to its “neighbors”, it is a less studied protein with only 380 categorized literature findings in the IKB. Lastly, vascular endothelial growth factor (VEGF) is a well established positive regulator of angiogenesis and is a downstream target of Bcl-2. Inhibition of vascular endothelial tube formation by Bcl-2 knockdown of BEAS-Cr and H460 cells (Figure 4B and C) supports the pro-angiogenic role of Bcl-2 which has not been directly demonstrated.

In conclusion, evidence presented in this report supports the role of Bcl-2 in Cr(VI)-induced lung tumorigenesis. Bcl-2 is upregulated in Cr(VI)-transformed cells and contributes to the apoptosis resistant phenotype of the cells. Such upregulation also contributes to the malignant properties of the cells including their invasiveness, angiogenicity, and anchorage-independent cell growth. Furthermore, animal studies support its role in tumorigenesis *in vivo*. Collectively, our data indicate the multifunctional role of Bcl-2 in the regulation of cell transformation and tumorigenesis in addition to its established role as an apoptosis regulator.
